# The therapeutic effect of ofatumumab in pediatric anti-NMDAR encephalitis: A case series

**DOI:** 10.1016/j.heliyon.2024.e40680

**Published:** 2024-11-23

**Authors:** Wenlin Wu, Jie Hong, Yanping Ran, Wenxiao Wu, Haixia Zhu, Chi Hou, Yuanyuan Gao, Yulin Tang, Yinting Liao, Wen-Xiong Chen, Xiaojing Li

**Affiliations:** Department of Neurology, Guangzhou Women and Children's Medical Center, Guangzhou Medical University, Guangzhou City, Guangdong Province, China

**Keywords:** Refractory, Anti-NMDAR encephalitis, Children, Ofatumumab

## Abstract

Ofatumumab (OFA) is an anti-CD20 antibody. We assessed the therapeutic potential of OFA in five pediatric anti-NMDAR encephalitis patients who showed poor responses to the first-line immunotherapy. OFA treatment showed clinical improvement including alleviation of clinical symptoms and mRS decrease accompanied by anti-NMDAR antibody turning negative in 3 patients and decline in 2 patients. And all patients achieved B cell depletion after OFA treatment. During follow-up, all patients’ symptoms were stable. OFA treatment is safe and effective, easy to administer, and favorable for pediatric anti-NMDAE encephalitis patients who are refractory to the first-line immunotherapy.

## Highlights

• Performance of a strategy of Ofatumumab (OFA) treatment in pediatric anti-NMDAE encephalitis patients.
• OFA treatment is effective, easy to handle, and favorable for pediatric anti-NMDAE encephalitis patients.
• OFA treatment is safe for pediatric anti-NMDAE encephalitis patients.

## 1 Introduction

Anti-N-methyl-D-aspartate receptor (NMDAR) encephalitis is a prevalent subtype of autoimmune encephalitis (AE), driven by a B-cell mediated immune response. This disease is characterized by the production of anti-NMDAR antibodies, which specifically target and bind to the NR1 subunit of the NMDAR receptor, resulting in pathological changes [[1], [2], [3]]. Anti-NMDAR encephalitis has a reported mortality rate of 2–5%. However, with timely and appropriate treatment, over 80 % of patients have the potential to make a significant recovery [4]. The International Consensus on treatment guidelines for pediatric anti-NMDAR encephalitis suggests that all affected children should receive corticosteroids, preferably administered as a pulsed intravenous infusion. For severe cases, adjunctive therapies such as intravenous immunoglobulin (IVIG) or plasma exchange (PE) are recommended [5]. For patients who are refractory to first-line treatments, second-line treatment are advised, with rituximab (RTX) being favored over cyclophosphamide [5]. About 10 %–30 % of pediatric anti-NMDAR encephalitis patients need second-line immunotherapy with RTX prefer to be used [[6], [7], [8], [9]]. RTX is an IgG1 mouse-human anti-CD20 monoclonal. B cell depletion therapies using RTX is effective for AE patients to achieve good outcome, decrease modified Rankin Scale (mRS) and relapse rate [10]. However, infusion related reactions are the most common adverse effect of RTX treatment, accounting for 15.7 % of AE [10].

Ofatumumab (OFA) is a fully human IgG1 monoclonal antibody targeting CD20, and it has been employed in the treatment of relapsing multiple sclerosis (MS) in adults [11]. Nevertheless, there are only a few case studies that explore its therapeutic efficacy in adult autoimmune encephalitis [[12], [13], [14]]. To date, little data have been available for the therapeutic effect of OFA in pediatric patients with anti-NMDAR encephalitis. Our study depicted five pediatric anti-NMDAR encephalitis patients receiving OFA treatment as the second-line immunotherapy.

## 2 Patients and methods

### 2.1 Patients

Pediatric patients (under 18 years old) who were diagnosed with anti-NMDAR encephalitis according to the criteria proposed by Graus et al. [15] and were treated with OFA as a second-line immunotherapy in the Neurology Department of Guangzhou Women and Children's Medical Center, Guangzhou Medical University, were included. Neurological disability was evaluated using the mRS. The disease severity was assessed by the clinical assessment scale for autoimmune encephalitis (CASE) derived from Lim et al. [16]. A poor treatment response was defined as no improvement on the mRS or mRS score ≥4 for 4 weeks [17].

### 2.2 NMDAR antibody testing

The NMDAR antibody testing in cerebrospinal fluid (CSF) and serum samples was conducted using a cell-based assay method, as previously described in our earlier publication [18]. Briefly, this method involves the transfection of HEK293 cells with the NR1 subunit of the NMDAR, followed by incubation with patient samples and detection using a fluorescent secondary antibody. The detailed protocol can be found in our previous study [18].

### 2.3 Treatment

The OFA treatment regimen was partially referred to the OFA treatment in the adult MS, in which OFA was administered by 20 mg subcutaneously 0, 1st, second and fourth week and every 4 weeks after that [19]. OFA used in anti-NMDAR encephalitis patients who had poor response to the first-line immunotherapy was administered 20 mg subcutaneously every week for the first three doses, and if there were adverse effects such as liver enzyme increase, the interval between doses was extended. If the B cell depletion was not achieved after the first three loading doses of OFA, the subsequent dose of OFA was administered.

## 3 Results

**Fig. 1:**
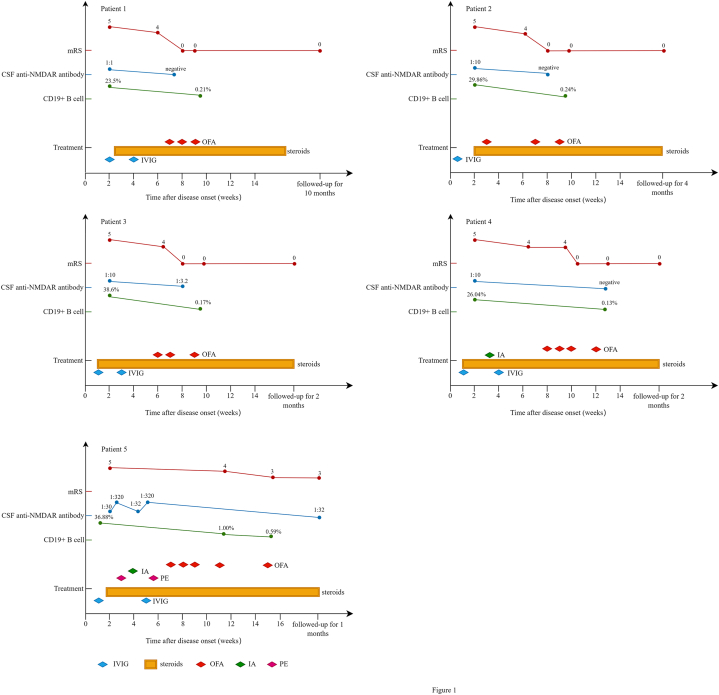
**Timeline depicting changes of mRS, CSF anti-NMDAR antibody, CD19**^**+**^**B cell percent, treatment in five patients.** The mRS scale is now indicated in points. The CD19^+^ B cells was measured in percentage. Blue diamond represents one course of IVIG. Red diamond represents one dose of OFA treatment. Green diamond indicates that IA treatment was used, though the number of cycles is not shown. Purple-red diamond indicates that PE treatment was used, with the number of exchanges not displayed. NMDAR, N-methyl-D-aspartate receptor; CSF, cerebrospinal fluid; mRS, Modified Rankin Scale; IVIG, intravenous immunoglobulin; PE, plasma exchange; OFA, Ofatumumab.

**Patient 1:** a 14-year-old female exhibited anti-NMDAR encephalitis with seizure, consciousness disturbance, memory deficit, agitation and behavioural changes. CSF analysis showed elevated white blood cells (WBC) and protein, and the anti-NMDAR antibody was 1:1. Serum anti-NMDAR antibody was negative. Electroencephalogram (EEG) showed slow waves with epileptic discharge and brain magnetic resonance image (MRI) showed small lesions in bilateral frontal subcortical white matter. No tumor was detected. The worst mRS was 5 and worst CASE was 14. The patient showed poor response to two courses of IVIG and intravenous methylprednisolone (IVMP) treatment. Seven weeks after onset, OFA treatment was initiated. Following OFA treatment, clinical improvements were observed, including cessation of seizures, the restoration of normal consciousness, resolution of agitation, and correction of memory deficits. CSF anti-NMDAR antibody became negative after the first dose of OFA treatment. Both mRS and CASE scores decreased to 0 after the final dose of OFA, with a significant reduction in the percentage of CD19^+^ B cells in peripheral blood lymphocytes. The patient remained stable during a 10-month follow-up period after the final dose of OFA (more details seen in Table 1 & Fig. 1).

**Table 1:** Clinical data of anti-NMDAR encephalitis patients treated with OFA.

Items	Patient 1	Patient 2	Patient 3	Patient 4	Patient 5
Age at onset (yrs)	14	12	11	11	11
Sex	Female	Male	Female	Female	Female
Tumor	None	None	None	None	A teratoma (17mm × 14 mm x 10 mm) in right adnexal area Underwent teraomectomy
CSF WBC count before OFA (x10^6^/L)	269	132	1	31	83
CSF protein before OFA (g/L)	0.75	0.37	0.14	0.40	0.37
Peripheral CD19^+^ B cells (%) before OFA	23.35	29.86	38.60	26.04	19.70
EEG before OFA	Slow background (1.5–3.0 Hz) with epileptic discharge	Slow background (8.0 Hz) with epileptic discharge	Slow background (5.0–6.0 Hz) with epileptic discharge	Slow background (1.5–2.0 Hz) with epileptic discharge	Slow background (1.0–2.5 Hz) with epileptic discharge
Brain MRI before OFA	Small lesions in bilateral frontal subcortical white matter	Normal	Lesions in bilateral periventricular white matter	Multiple demyelination lesions in the bilateral frontal lobe, temporal lobe and parietal lobe	Lesions in the bilateral globus pallidus, caudate nucleus, frontal lobe, temporal lobe and insular cortex
CSF anti-NMDAR antibody titer before OFA	1:1	1:10	1:10	1:30	1:320
Serum anti-NMDAR antibody titer before OFA	Negative	Negative	Negative	Negative	1:1000
mRS before OFA	5	5	5	5	5
CASE before OFA	14	17	16	22	24
Immunotherapy before OFA	IVMP; IVIG	IVMP; IVIG	IVMP; IVIG	IVMP; IVIG; IA	IVMP; IVIG; PE; IA
Antiepileptic drugs	N/A	N/A	LEV; NZP; VPA	LCM; CXZ	LEV; CXZ
Psychotropic medications	Risperidone	Risperidone	Risperidone	Risperidone	Risperidone
Duration from onset to OFA treatment (weeks)	7	3	6	8	9
Number of OFA infusions	3	4	3	4	5
CSF WBC count after OFA (x10^6^/L)	7	1	3	7	8
Peripheral CD19^+^ B cells (%) after OFA	0.21	0.24	0.17	0.13	0.56
EEG after OFA	Background activity: left normal, right diffuse slowing (1.5–3 Hz); Reduced epileptic discharge	Normal background with occasional slow waves; No epileptic discharge	Asymmetric abnormal background activity: left posterior temporal slowing, right occasional occipital slowing; Reduced epileptic discharge	Asymmetric background, poor rhythm in right occipital region; Reduced epileptic discharge	Slow background (3–4 Hz) without epileptic discharge
Brain MRI after OFA	Lesions resolved	N/A	No change	Lesions reduced	Lesions resolved
CSF anti-NMDAR antibody titer after OFA	Negative	Negative	1:3.2	Negative	1:32
Serum anti-NMDAR antibody titer after OFA	N/A	N/A	N/A	N/A	1:320
mRS After OFA	0	0	0	0	3
CASE after OFA	0	0	0	0	10
Side effect of OFA	None	Mild allergic reactions & liver enzyme increased	Liver enzyme increased	None	None
Duration of follow-up (months)	10	4	2	2	1

CSF, cerebrospinal fluid; CASE, clinical assessment scale for autoimmune encephalitis; CXZ, Clonazepam; EEG, electroencephalogram; IA, immunoadsorption; IVIG, intravenous immunoglobulin; IVMP, intravenous methylprednisolone; LCM, Lacosamide; LEV, Levetiracetam; MRI, magnetic resonance image; mRS, Modified Rankin Scale; NZP, Nitrazepam; NMDAR, N-methyl-D-aspartate receptor; N/A, not applicable; OFA, Ofatumumab; PE, plasma exchange; VPA, valproic acid; WBC, white blood cell counts.

**Patient 2:** A 12-year-old male exhibited anti-NMDAR encephalitis with high-frequent seizures occurring every 2–3 minutes, leading to status epilepticus that required invasive mechanical ventilation and continuous intravenous administration of midazolam. The patient also presented with consciousness disturbance, memory deficit, agitation, and behavioral changes. CSF analysis showed elevated WBC with normal protein levels, and the anti-NMDAR antibody titer was 1:10. The serum anti-NMDAR antibody was negative. EEG showed slow waves with epileptic discharge and brain MRI was normal. No tumor was detected. The worst mRS was 5 and worst CASE was 17. After one course of IVIG and IVMP treatment, the patient was successfully weaned off the ventilator, but experienced a seizure. Despite this, the psychiatric symptoms and cognitive impairment did not improve.

Three weeks after onset, OFA treatment was initiated. Two days after the first dose of OFA treatment, the patient presented with hyperventilation and chest computed tomography showed uneven aeration of both lung fields and a mosaic attenuation pattern was observed in some lung regions, which suggested the possibility of small airway disease. Hyperventilation was considered a manifestation of an allergic reaction. Furthermore, serum alanine aminotransferase (ALT) increased to 260 U/L. The patient inhaled budesonide with salbutamol and received oral antihistamines and hepatoprotective agents. Seven weeks after onset, breathing and ALT levels normalized. OFA treatment continued with 20 mg every two weeks for 4 weeks. After undergoing treatment with OFA, the patient exhibited significant clinical improvement, characterized by resolution of agitation correction of memory deficits. The CSF anti-NMDAR antibody became negative after the second dose of OFA treatment. Both mRS and CASE scores decreased to 0 after the final dose of OFA, with a significant reduction in the percentage of CD19^+^ B cells in peripheral blood lymphocytes. The patient remained stable during the four-month follow-up period after the final dose of OFA (more details seen in Table 1 & Fig. 1).

**Patient 3:** An 11-year-old female exhibited anti-NMDAR encephalitis with seizure, consciousness disturbance, severe insomnia, agitation, behavioural changes, a decrease of verbal output, nystagmus and urinary and bowel disorders requiring an indwelling urinary catheter. CSF analysis showed normal WBC and protein levels, with an anti-NMDAR antibody titer of 1:10. Serum anti-NMDAR antibody was negative. EEG showed slow waves with epileptic discharge and brain MRI showed lesions in bilateral periventricular white matter. No tumor was detected. The worst mRS was 5 and worst CASE was 16. After two courses of IVIG and IVMP treatment, the patient became seizure-free, but the other symptoms persisted.

Six weeks after onset, OFA treatment was initiated with a dose of 20 mg subcutaneously 0 and first week. After the second dose of OFA, liver enzyme ALT increased to 136 U/L and normalized within two weeks following treatment with oral hepatoprotective agent. Nine weeks after onset, OFA treatment continued with 20 mg once. After undergoing treatment with OFA, the patient exhibited significant clinical improvement, including the resolution of agitation and nystagmus, restoration of normal sleep, and successful removal of the urinary catheter. The CSF anti-NMDAR antibody decreased to 1:3.2 after the second dose of OFA treatment. Both mRS and CASE scores decreased to 0 after the final dose of OFA, with a significant reduction in the percentage of CD19^+^ B cells in peripheral blood lymphocytes. The patient remained stable during the two-month follow-up period after the final dose of OFA (more details seen in Table 1 & Fig. 1).

**Patient 4:** An 11-year-old female exhibited anti-NMDAR encephalitis with frequent seizure (focal seizures occurring every 10–20 minutes and generalized), consciousness disturbance, severe insomnia, agitation, behavioural changes, a decrease of verbal output, dyskinesias, dystonia and urinary and bowel disorders requiring an indwelling urinary catheter. CSF analysis showed elevated WBC with normal protein levels, and anti-NMDAR antibody titer was 1:10. The serum anti-NMDAR antibody was negative. EEG showed slow waves with epileptic discharge and brain MRI showed multiple demyelination lesions in the bilateral frontal lobe, temporal lobe and parietal lobe. No tumor was detected. The worst mRS was 5 and worst CASE was 22.

After one course of IVIG and IVMP treatment, the patient still had frequent seizures and consciousness worsened (Glasgow Coma Scale decreased to 9 points). Protein A immunoadsorption (IA) was initiated, with four sessions every other day, followed by a second IVIG course. After that, the patient became seizure-free and Glasgow Coma Scale improved to 11, but other symptoms remained unchanged.

Eight weeks after onset, OFA treatment was initiated with the dose of 20 mg subcutaneously at weeks 0, 1, 2, and 4. After undergoing treatment with OFA, the patient exhibited significant clinical improvement, including the resolution of agitation, dyskinesis and dystonia, restoration of normal sleep, the ability to engage in simple communication and successful removal of the urinary catheter. The CSF anti-NMDAR antibody became negative. Both mRS and CASE scores decreased to 0 after the final dose of OFA and brain MRI lesions were partly absorbed, with a significant reduction in the percentage of CD19^+^ B cells in peripheral blood lymphocytes. The patient remained stable during the two-month follow-up period after the final dose of OFA (more details seen in Table 1& Fig. 1).

**Patient 5:** An 11-year-old female exhibited anti-NMDAR encephalitis with behavioural changes, consciousness disturbance, frequent seizure occurring about every one to 2 h, agitation, a decrease of verbal output, dyskinesias, dystonia and urinary and bowel disorders requiring an indwelling urinary catheter. CSF analysis showed elevated WBC with normal protein, and the anti-NMDAR antibody titer was 1:30. CSF next-generation sequencing identified 59 sequences for the human herpes virus 4 and 3 sequences for human herpes virus 6B. EEG showed slow waves with epileptic discharge and brain MRI showed lesion in the bilateral globus pallidus, caudate nucleus, frontal lobe, temporal lobe and insular cortex. The worst mRS was 5 and worst CASE was 24.

After two courses of IVIG and IVMP treatment and acyclovir antiviral therapy, the CSF WBC increased to 83x10^6^/L, and the CSF anti-NMDAR antibody titer increased to 1:320, while the serum anti-NMDAR antibody titer was 1:3200. The patient developed more frequent seizure and Staphylococcus aureus pneumonia requiring invasive mechanical ventilation. No tumor was detected by abdomen and pelvis computed tomography, and the patient's vital signs were unstable for pelvis MRI examination. PE was initiated with four daily sessions. After PE, the patient became seizure-free, but other symptoms persisted, and both serum and CSF anti-NMDAR antibody titer remained unchanged.

IA was subsequently initiated with four sessions on days 1, 2, 4 and 5. Following IA, the patient was weaned from the ventilator and the CSF WBC normalized. CSF anti-NMDAR antibody titer decreased to 1:32, and serum titer dropped to 1:100. Despite these improvements, consciousness disturbance, agitation, dyskinesias, dystonia and urinary issues persisted. One week after IA, seizures recurred, accompanied by rhabdomyolysis and intermittent heart rate dropped (20–60 bpm). CSF and the serum anti-NMDAR antibody titer increased to 1:320 and 1:1000, respectively.

PE was re-administered with four daily sessions. Afterward, seizure and rhabdomyolysis resolved, but other symptoms persisted. Nine weeks after onset, OFA treatment was initiated with 20 mg at weeks 0, 1, 2, and 4. Following the fourth OFA dose, the patient showed gradual improvement, including reduction in dyskinesias and dystonia, resolution of agitation, and the restoration of nighttime sleep and ability to engage in simple communication. Brain MRI lesions resolved. A teratoma (17 mm × 14 mm x 10 mm) was found in the right adnexal area and surgically removed. One month after the fourth OFA dose, a fifth dose was administered, resulting in mRS score reduction to 3 and CASE score reduction to 10, with a significant reduction in the percentage of CD19^+^ B cells in peripheral blood lymphocytes. The CSF and the serum anti-NMDAR antibody titer decreased to 1:32 and 1:320, respectively. The patient remained stable during the one-month follow-up period (more details seen in Table 1& Fig. 1).

## 4 Discussion

Anti-NMDAR encephalitis is the most common type of AE. The first-line immunotherapy for anti-NMDAR encephalitis included steroids, IVIG or PE. When the response to the first-line therapy is inadequate or when the disease is severe or relapsing, escalation of immunotherapy to the second-line immunotherapy is needed [5,20]. Considering its better tolerance, fewer side effects, and independence from plasma availability, IA is also used as a viable option for severe patients in clinical practice to address plasma shortages and reduce the potential complications associated with PE [[21], [22], [23]]. About 10 %–30 % of pediatric anti-NMDAR encephalitis patients need second-line immunotherapy with RTX prefer to be used [[6], [7], [8], [9]].

RTX is the first monoclonal anti-CD20 antibody licensed for use against human disease. The CD20 is expressed on pre-B-cells from an early development stage but not on the precursor hematopoietic stem cells and antibody-secreting plasma cells [24].Anti-NMDAR encephalitis is B-cell mediated AE with an anti-NMDAR antibody as its actual pathogenic antibody [2]. B cells can produce anti-NMDAR antibodies causing NMDAR internalized and leading to NMDAR hypofunction, extrasynaptic NMDAR hyperfunction or neuronal network imbalance with impaired intraneuronal activity which is involved in the pathological mechanism of anti-NMDAR encephalitis [3,25]. In addition, B cells can secrete cytokines modulating various immune responses and serve as highly effective antigen-presenting cells [26,27]. B cell depletion therapies using RTX are effective for AE patients to achieve good outcomes and decrease mRS and relapse rates (10).

In the context of B-cell depletion therapies, both anti-NMDAR encephalitis and MS share a therapeutic approach involving the use of agents like RTX. In anti-NMDAR encephalitis, B-cell depletion therapies, such as RTX, serve a dual role. They are employed not only for managing acute episodes, particularly in cases where patients respond poorly to first-line immunotherapy, but also as maintenance therapy to prevent further relapses [[6], [7], [8], [9]]. In contrast, in MS, B-cell depletion therapies are primarily utilized as second-line disease-modifying therapies. These therapies are initiated early in the disease course, often at diagnosis, with the primary objective of reducing relapse frequency and slowing the progression of disability. Unlike in anti-NMDAR encephalitis, B-cell depletion in MS is not typically used for acute relapse management, which is generally administered with high-dose corticosteroids. In pediatric-onset MS, RTX is one of the most commonly used second-line immunosuppressive therapies, reflecting its effectiveness in cases where first-line treatments are insufficient [28,29].

However, infusion-related reactions are the most common adverse effect of RTX treatment, accounting for 15.7 % of AE [10]. RTX is administered intravenously and necessitates pretreatment with antihistamines and steroids, which can be challenging for anti-NMDAR encephalitis patients exhibiting severe psychiatric symptoms. For these reasons, it is necessary to explore alternatives that provide a better balance of safety, efficacy, and ease of administration.

Different from RTX, both Ocrelizumab and OFA are humanized anti-CD20 monoclonal antibodies making them less likely to cause allergic reactions [30]. Ocrelizumab, the first anti-CD20 mAb approved for the treatment of adults with relapsing MS or primary progressive MS, is administrated intravenously as two 300-mg infusions 14 days apart followed by 600 mg every 24 weeks [31,32]. The long treatment intervals of Ocrelizumab can reduce the need for frequent injections. However, Ocrelizumab is not available on the Chinese market. OFA was approved for use in treating adults with relapsing MS in the USA, across Europe and in several other countries [11]. The Phase III clinical trials of OFA, ASCLEPIOS I/II, demonstrated that patients receiving OFA treatment had lower ARR than those on teriflunomide. Additionally, OFA outperformed teriflunomide on Expand Disability Status Scale score worsening, magnetic resonance image metrics and serum neurofilament light chain concentration [19]. OFA, available on the Chinese market since December 2021 share similarities with RTX in that it can bind CD20, cause compartmentalization into lipid rafts, and recognize an epitope comprising both extracellular loops but binding closer to the cell membrane than RTX [24]. OFA efficiently depletes B cells through complement-dependent cytotoxicity and antibody-dependent cell-mediated cytotoxicity, even when CD20 expression is low [33]. The subcutaneous administration of OFA, delivered into the hypodermis, may lead to more efficient and selective targeting of B cells residing in the lymphatic circulatory system than intravenous administration directly entering the systemic circulation [34]. In previous studies, OFA has shown activity against RTX resistant chronic lymphocytic leukemia in vivo [35,36]. These findings suggest the effect of B cell depletion of OFA was at least as effective as RTX. OFA can be easily administered via subcutaneous injection. In clinical practice, it is often challenging for anti-NMDAR encephalitis patients with psychiatric symptoms such as agitation, aggression, dyskinesias, dystonia, or frequent seizures to complete RTX infusions. OFA, however, is highly convenient in these situations. Considering the safety, efficacy, ease of administration, and availability of OFA, we opted for OFA treatment in the five patients included in this study who were refractory to first-line immunotherapy.

In our study, three patients (patients 1, 2 and 3) were refractory to the first-line immunotherapy with IVIG and IVMP. Additionally, one patient (patient 4) showed poor response to IVIG, IVMP and IA, while another (patient 5) responded poorly to IVIG, IVMP, PE and IA. All five patients received OFA treatment as second immunotherapy. After OFA treatment, 4 patients (1, 2, 3, 4) showed remarkable clinical improvement, and the mRS decreasing to 0. One patient's (patient 5) status gradually improved, with mRS decreasing from 5 to 3. After OFA treatment, CSF anti-NMDAR antibody became negative in three patients and decreased in two patients. All patients achieved B cell depletion. These results suggested OFA effectively improved clinical symptoms and mRS and achieved B cell depletion in anti-NMDAR encephalitis. Recent studies, including a report from China, have demonstrated promising effects of OFA in treating adult patients with refractory anti-NMDAR encephalitis [[12], [13], [14]]. These effects of OFA in our anti-NMDAR encephalitis patients were similar to other studies about OFA treatment in adult AE. In total, OFA was an effective treatment for anti-NMDAR encephalitis patients who were refractory to the first-line immunotherapy.

The most common adverse effect of OFA treatment was injection-related reaction, most occurring during the first injection [19]. In our study, one patient had mild allergic reactions and liver enzyme increased after the first dose of OFA, and one patient had liver enzyme increased after the second dose of OFA. These allergic reactions and liver enzyme increase were improved after symptomatic treatment and did not occur in the subsequent dose of OFA, whether the dose of OFA and the interval between each must be adjusted in pediatric patients requiring further research.

There are several limitations in our study. The sample size was small without relapsed anti-NMDAR encephalitis patients, and the follow-up time was not long enough. Therefore, large samples with both initial onset and relapsed anti-NMDAR encephalitis patients and long follow-up duration are needed to definite the effect of OFA treatment in the future.

## 5 Conclusions

OFA treatment is safe and effective, easy to administer, and favorable for pediatric anti-NMDAR encephalitis patients who are refractory to the first-line immunotherapy.

## CRediT authorship contribution statement

**Wenlin Wu:** Writing – original draft, Investigation, Funding acquisition, Formal analysis, Data curation, Conceptualization. **Jie Hong:** Resources, Project administration, Methodology, Formal analysis, Data curation, Conceptualization. **Yanping Ran:** Resources, Project administration, Methodology, Investigation, Formal analysis, Data curation. **Wenxiao Wu:** Resources, Project administration, Methodology, Investigation, Formal analysis. **Haixia Zhu:** Validation, Supervision, Resources, Conceptualization. **Chi Hou:** Visualization, Validation, Supervision, Software, Resources, Funding acquisition. **Yuanyuan Gao:** Visualization, Validation, Supervision, Conceptualization. **Yulin Tang:** Validation, Resources, Conceptualization. **Yinting Liao:** Validation, Resources, Conceptualization. **Wen-Xiong Chen:** Writing – review & editing, Project administration. **Xiaojing Li:** Writing – review & editing, Project administration, Methodology, Investigation, Funding acquisition, Conceptualization.

## Ethics statement

This study was approved by the Ethics Committee of Guangzhou Women and Children Medical Center. Written informed consent was obtained from the patients’ parents or legal guardians for the publication of any potentially identifiable images or data included in this article.

## Data availability

The original contributions presented in the study are includedin the article. Further inquiries can be directed to the corresponding author.

## Funding

This work was supported by the Guangzhou Municipal Science and Technology Basic and Applied Research Project (Grant No. 202201020619), Applied Basic Research Foundation of Guangzhou City (Grant No.202102020252), Guangzhou Municipal Science and Technology Basic and Applied Research Project (Grant No.202201011838). The authors had full control of the data and information submitted for publication.

## Declaration of competing interest

The authors declare the following financial interests/personal relationships which may be considered as potential competing interests:

Xiaojing Li reports financial support was provided by Guangzhou Municipal Science and Technology Basic and Applied Research Project. Wenlin Wu reports financial support was provided by Applied Basic Research Foundation of Guangzhou City. Chi Hou reports financial support was provided by Guangzhou Municipal Science and Technology Basic and Applied Research Project. If there are other authors, they declare that they have no known competing financial interests or personal relationships that could have appeared to influence the work reported in this paper.

## Abbreviation

AE: autoimmune encephalitis
ALT: alanine aminotransferase
CASE: clinical assessment scale for autoimmune encephalitis
CSF: cerebrospinal fluid
EEG: electroencephalogram
IA: immunoadsorption
IVIG: intravenous immunoglobulin
IVMP: intravenous methylprednisolone
MRI: magnetic resonance image
mRS: Modified Rankin Scale
MS: multiple sclerosis
NMDAR: N-methyl-D-aspartate receptor
OFA: Ofatumumab
PE: plasma exchange
RTX: rituximab
WBC: white blood cell counts
